# The osteoinductive potential of different root-end filling materials in a rat femur model

**DOI:** 10.1038/s41598-024-52584-5

**Published:** 2024-01-24

**Authors:** Seçkin Aksu, Ebru Delikan, Ayşe Özcan Küçük, Zehra Demiray Asoğlu, Şakir Necat Yılmaz

**Affiliations:** 1 Department of Pediatric Dentistry, Faculty of Dentistry, Mersin University, Mersin, Turkey; 2 Department of Pediatric Dentistry, Faculty of Dentistry, Nuh Naci Yazgan University, Kayseri, Turkey; 3 Department of Oral and Maxillofacial Surgery, Faculty of Dentistry, Mersin University, Mersin, Turkey; 4 Department of Hıstology and Embryology, Faculty of Medicine, Hatay Mustafa Kemal University, Hatay, Turkey; 5 Department of Hıstology and Embryology, Faculty of Medicine, Mersin University, Mersin, Turkey

**Keywords:** Biochemistry, Cell biology, Computational biology and bioinformatics, Developmental biology, Microbiology, Structural biology, Systems biology, Biomarkers, Medical research, Materials science

## Abstract

In pediatric dentistry, the enduring success of root-end filling materials employed for the establishment of apical plugs in immature teeth undergoing endodontic intervention is contingent upon their possessing a robust osteoinductive capacity. Hence, the primary objective of this study was to histologically assess the osteoinductive potential of four distinct dental materials, specifically recommended for retrograde filling applications, utilizing an animal hard tissue model. Within the designed experimental model, two bone defects measuring 2 mm in diameter and 2 mm in depth were created in both femurs of a cohort comprising 21 male Wistar albino rats. The first defect in the right femur was left blank as the control group, and Neo MTA Plus was placed in the second defect. The EndoSequence BCRRM Fast Set Putty was placed in the first of the defects on the left femur, and Endo Repair was placed in the second defect. Subjects were sacrificed after 7, 14, and 28 days of follow-up, and sections were examined to assess the degree of inflammation, connective tissue formation, and new bone formation. The data were statistically evaluated with Kruskal‒Wallis and post hoc Dunn's tests using SPSS 12 software. The bone healing levels of the Neo MTA Plus group were significantly higher than those of the other groups in all periods (p < 0.05). Bone organization in all groups decreased over time, and fibrous tissue was enriched. The results of this study demonstrate that NeoMta Plus has superior osteoinductive properties compared to other materials but that EndoSequence and Endo Repair have the potential to be developed.

## Introduction

Dental trauma resulting in pulp injury frequently occurs in recently erupted or partially developed permanent teeth. However, inadequate oral hygiene can lead to caries with incomplete maturation and large pulp chambers, potentially resulting in infections and arrested root development. Arrested root development can cause the apex to be too wide, complicating conventional endodontic treatment^1^.

Root canal treatment of immature teeth requiring endodontic treatment can be achieved in a single session by forming an apical plug and using root-end filling materials. This is a viable alternative to the traditional multisession apexification procedure. The introduction of new biocompatible and highly impermeable root-end filling materials, which cause healthy complex tissue formation and minimal inflammation in the apical area, has increased the success rate of apexification treatment^2,3^.

Mineral trioxide aggregate (MTA), which has been successfully used in endodontic treatments in pediatric dentistry, consists of hydrophilic residues such as tricalcium silicate, dicalcium silicate, tricalcium aluminate, calcium sulfate dihydrate, and bismuth oxide. The most important physical property of this biocompatible and antibacterial material is its impermeability to moisture^4,5^. There is also evidence that MTA stimulates hard-tissue formation^6^. Despite its various beneficial features, the use of this biomaterial is limited by factors such as lengthy initial setting time, complexity of its clinical application owing to its sandy nature, need for additional moisture, and inability to maintain condensation pressure during barrier formation. To overcome these disadvantages, the development of biomaterials with shorter curing times, better biocompatibility, and easier clinical use has accelerated^7,8^.

NeoMTA Plus (nMTA), a derivative of MTA, has been developed to circumvent the disadvantages of MTA. Owing to the fine particles in the powder, it is easier to mix, and its liquid retains moisture for a longer time, preventing drying and providing a smooth and soft mixture^9^. Additionally, nMTA hardens faster than MTA, thereby reducing chair time and the number of sessions, which is especially beneficial for pediatric patients^10^.

EndoSequence BC RRM Fast Set Putty (ERRM), a calcium silicate-based product, has been marketed as a biocompatible and bioactive material with a good sealing capacity^11^. It is designed to be easier to use clinically because of its syringable form while also providing enhanced color stability. Furthermore, their physical and chemical properties are comparable to those of MTA. An additional benefit of ERRM is that it releases calcium and phosphate ions, which are necessary for hydroxyapatite deposition^12^. It has also demonstrated similar compressive strength to ProRoot MTA, as well as antibacterial and antifungal activities^13^.

Endo Repair (ER) is a sealing agent composed of calcium phosphate and hydroxyapatite in powder form and distilled water in liquid form. It is used in vital pulpotomy treatments after direct and indirect pulp capping, apexification treatments, root perforations, deep caries, or iatrogenic pulp injuries. The manufacturer claims that the material is easy to use, has a short setting time, and does not cause necrosis^14^. However, there has been limited research concerning this material in the literature, with none relating to its osteogenic potential^15,16^.

Due to the paucity of in vitro studies on ER, a dental material investigated in this study, an animal model was included as an initial study before initiating clinical studies. Ethically approved rats for research in our country were selected considering cost effectiveness, time efficiency, minimal risk of postoperative infection, genetic homogeneity, and facilitation of comparative analysis of tissue responses in the same animal^17,18^. The aim of this study was to histologically evaluate the osteoinductive potential of nMTA, ERRM, and ER in stimulating hard tissue formation in rat femur models. The evaluation of these materials, which have not been compared with each other in vivo before determining an easy-to-apply and cost-effective retrograde filling material for dentists and patients, constitutes the original value of our study. The null hypothesis of the study is that the materials tested in this study are not able to repair and regenerate bone when applied to cavities created in femoral rat models and that there is no difference between them with respect to their osteoinductive properties.

## Materials and methods

This study was approved by the Ethics Committee of Experimental Animals of Mersin University (Approval No. 2018/52602694). This study was performed in accordance with the principles of the accordance with ARRIVE guidelines related to the protection of laboratory animals. The sample size of the three independent groups was calculated a priori using power analysis (Test power, 0.80; effect size, 0.684; significance level, 0.05). A minimum sample size of seven was calculated for each subgroup. According to this analysis, 21 rats were used in our study. Twenty-one male Wistar albino rats (4–5 months and weighing 200–250 g) were obtained from the Experimental Animal Research Unit of Mersin University, Turkey, and the experimental procedures were performed at the same center. To acclimatize the animals to their environment, they were kept in a cycle of 12 h of darkness and 12 h of light at a temperature of 21 °C with ad libitum access to food and water.

### Surgical procedures

Twenty-one male Wistar Albino rats were included in this study. Rats were anesthetized with intramuscular injections of ketamine hydrochloride (Ketamidor-Richet Pharma, Wels, Austria, 50 mg/kg) and xylazine hydrochloride (Rompun-Bayer, Rompun, Seoul, Korea, 5 mg/kg). The operation area of each rat was prepared by shaving with a razor blade and disinfecting with an iodine solution skin disinfectant. Following the induction of general anesthesia, local infiltration anesthesia was administered with 4% articaine hydrochloride containing 0.006 mg/mL epinephrine (Ultracain D-S Forte-Aventis, Istanbul, Turkey) in the operative area for local hemostasis.

A 15-mm medial straight incision was made along the longitudinal axis of the right and left femurs with a sterile no. 15 scalpel to expose the surgical area and generate a midline defect in the rat femoral bone. Retractors were used to ensure adequate visibility in the surgical field, and two defects with a diameter and depth of 2 mm were created in the right and left femurs of each patient using a tungsten carbide bur with a diameter of 2 mm under continuous irrigation with 0.9% saline solution (G-Biosciences, Missouri, USA). Animal surgical procedures were performed by the same experienced surgeon to maintain high consistency.

The first defect was left empty on the right femur as the control group, whereas nMTA (Avalon Biomed Inc, Bradenton, FL, USA) was placed in the second. The first defect on the left femur was filled with ERRM (Brasseler, Savannah, Georgia, USA), and the second was filled with ER (Hoffmann Dental Manufaktur GmbH, Berlin, Germany). The materials used in this study are listed in Table 1.

**Table 1:** Experimental materials used in the study.

Material	Composition	Manufacturer
nMTA	Portland cement (Tricalcium Silicate, Tricalcium Aluminate, Dicalcium Silicate, Tetra Calcium Alumina Ferrite), Tantalum Oxide	Avalon Biomed Inc, Bradenton, FL
ERRM	Calcium silicates, Zirconium oxide, Tantalum oxide, Calcium phosphate	Brasseler, USA
ER	Calcium phosphate, Hydroxyapatite, Distilled Water	Hoffmann Dental Manufaktur GmbH, Germany

Following the completion of the procedure, the operating sites were primarily closed using Vicryl 3/0 sutures (Ethicon, Florida, USA). Hemostasis was achieved by applying a sterile tampon. The rats were then transferred to Type IV Euro standard cages and monitored under standard conditions. After surgery, to prevent postoperative infection, an intramuscular injection of 30 mg/kg ceftriaxon (Roche Diagnostics, Basel, Switzerland) and 4 mg/kg carprofen (Pfizer, Inc., New York, NY, USA) was administered to the animals every 24 h for three days starting immediately following the surgery and were allowed to move freely in the cages with access to standard rat chow and tap water. Wound healing, incision sites, activity, and food consumption were monitored daily by the surgeon, two dentists possessing certificates for experimental animals, and a veterinarian to avoid post-traumatic fractures. No unexpected death and emergency euthanasia were required during the entire experiment.

The subjects were randomly divided into three groups and seven animals each euthanized with an overdose of pentobarbital (Bioveta, Ivanovice na Hané, Çekya 500 mg/kg) on days 7, 14, and 28. Femoral bone samples (84 sample) were carefully removed from the defect area at a distance of 3 mm using burs. For histological analysis, the femurs were fixed in 10% formalin solution (Sigma-Aldrich, Missouri, USA).

### Histological examinations

The samples were fixed in 10% neutral-buffered formalin (Sigma-Aldrich, Missouri, USA) and subsequently decalcified with ethylenediaminetetraacetic acid (Sigma-Aldrich, Missouri, USA). Decalcified tissues were embedded in paraffin blocks using the standard light microscopic evaluation method. Using a microtome (Leica Rotary Microtome RM2125, IL 60010 United States), 7-μm-thick slides were obtained from the samples at the levels where the defects were seen at full width. Skipping ten slides and taking one of them, five slides were evaluated for each sample. Slides were stained with Hematoxylin and eosin (H&E) (Sigma-Aldrich, Missouri, USA). Slides were photographed under a light microscope (Olympus BX50, Tokyo, Japan) and evaluated blindly by a histologist. All sections were histologically evaluated and photographed under a light microscope with 400X magnification. The scoring was performed by a single histologist at one time.

The bone healing levels of the defects were evaluated and scored on a scale of 1–5^19,20^.

**Score 1:** Necrotic tissue or no sign of improvement in the defect.

**Score 2:** Inflammation and/or fibrosis in the defect area.

**Score 3:** Presence of new cartilage formation in the defect area but no new bone formation.

**Score 4:** Immature bone formation in addition to new cartilage production in the defect area.

**Score 5:** Newly formed bone tissue acquired mature compact bone properties.

**Figure 1:**
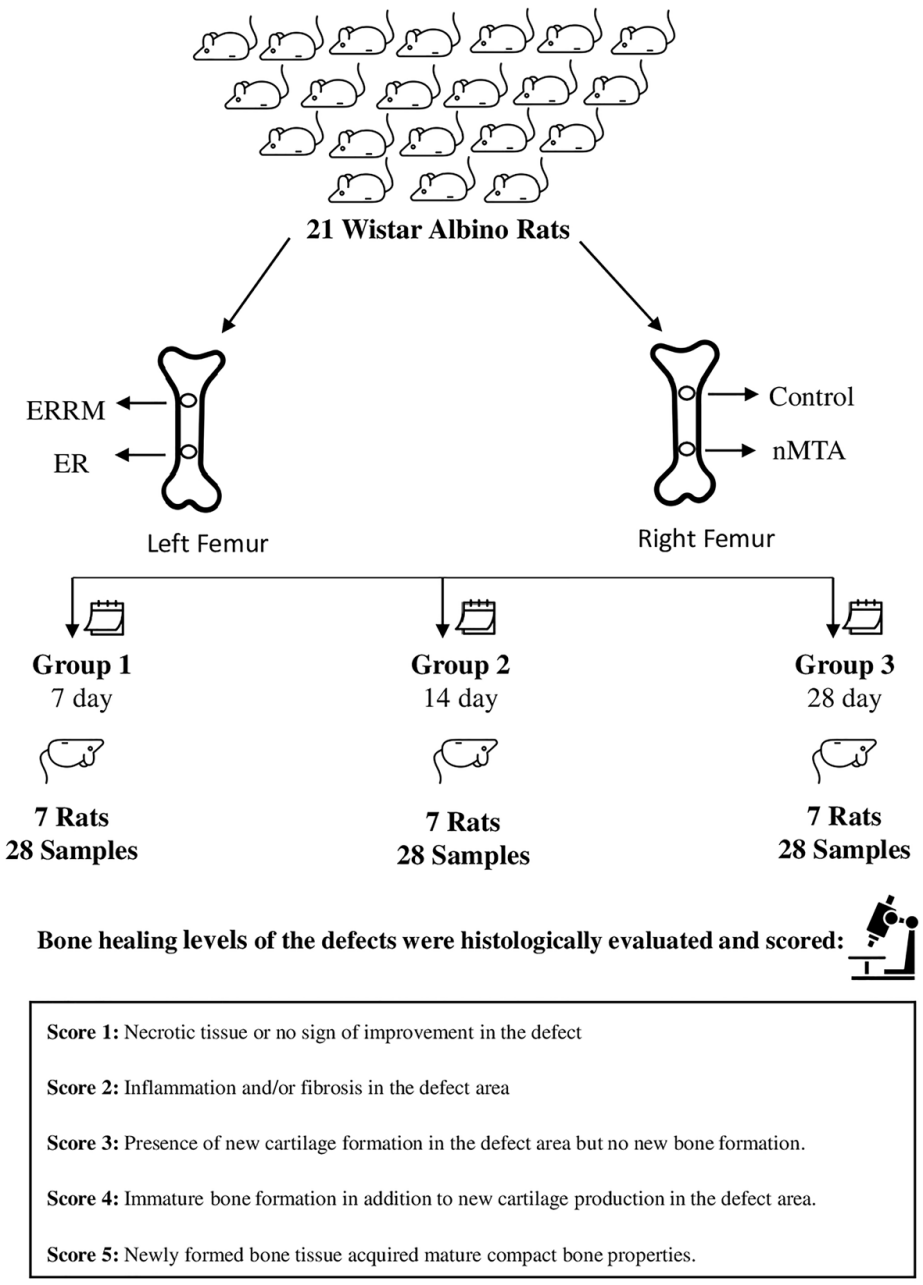
Flowchart of experimental procedure.

A flowchart or diagram of the experimental procedure is represented in Fig. 1.

### Statistical analysis

Statistical analysis was conducted using Statistical Package for the Social Sciences version 22 (Windows, SPSS Inc., Chicago, IL, USA). To analyze non-parametric data, the Kruskal‒Wallis test and post hoc Dunn’s test were applied, with statistical significance set at p < 0.05.

## Ethical approval and consent to participate

This study was approved by the Ethics Committee of Experimental Animals of Mersin University (Approval No. 2018/52602694). All methods were performed in accordance with the relevant guidelines and regulations.

## Results

**Figure 2:**
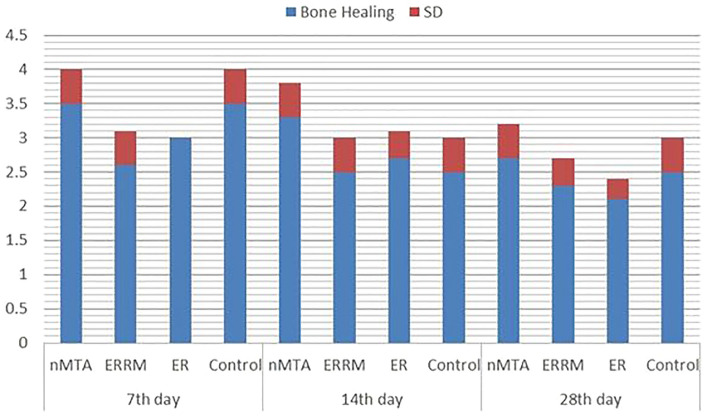
Distribution of bone healing scores of materials on different days.

**Figure 3:**
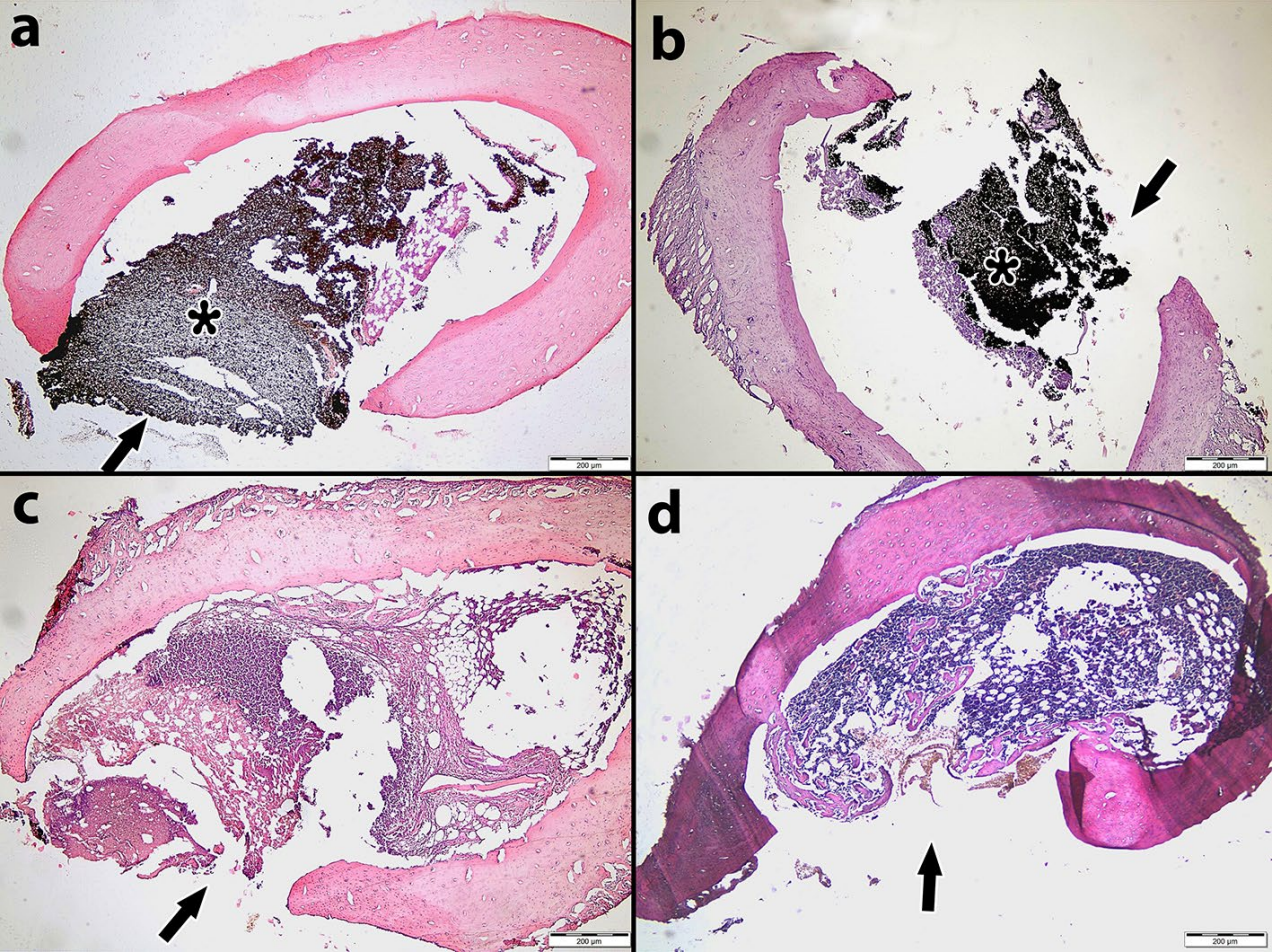
Histological sections of rat femur bone stained with Hematoxylin–Eosin on the 7th day. (**a**) nMTA group; The filling material has not been removed and there is no shrinkage in the defect area. (**b**) ERRM group; The filling material was not removed and no shrinkage of the defect area was observed. There is granulation tissue in the medulla of the bone. (**c**) ER, no filling material was observed, granulation tissue formation was observed in the medulla. (**d**) Control group; There is dense granulation in the medulla of the rat femur bone. There is no closure in the defect area. (Magnification bar = 200 µm, H&E 400 ×). Arrow: Defect area, Asterisk: Root end-filling material. (n = 7).

**Figure 4:**
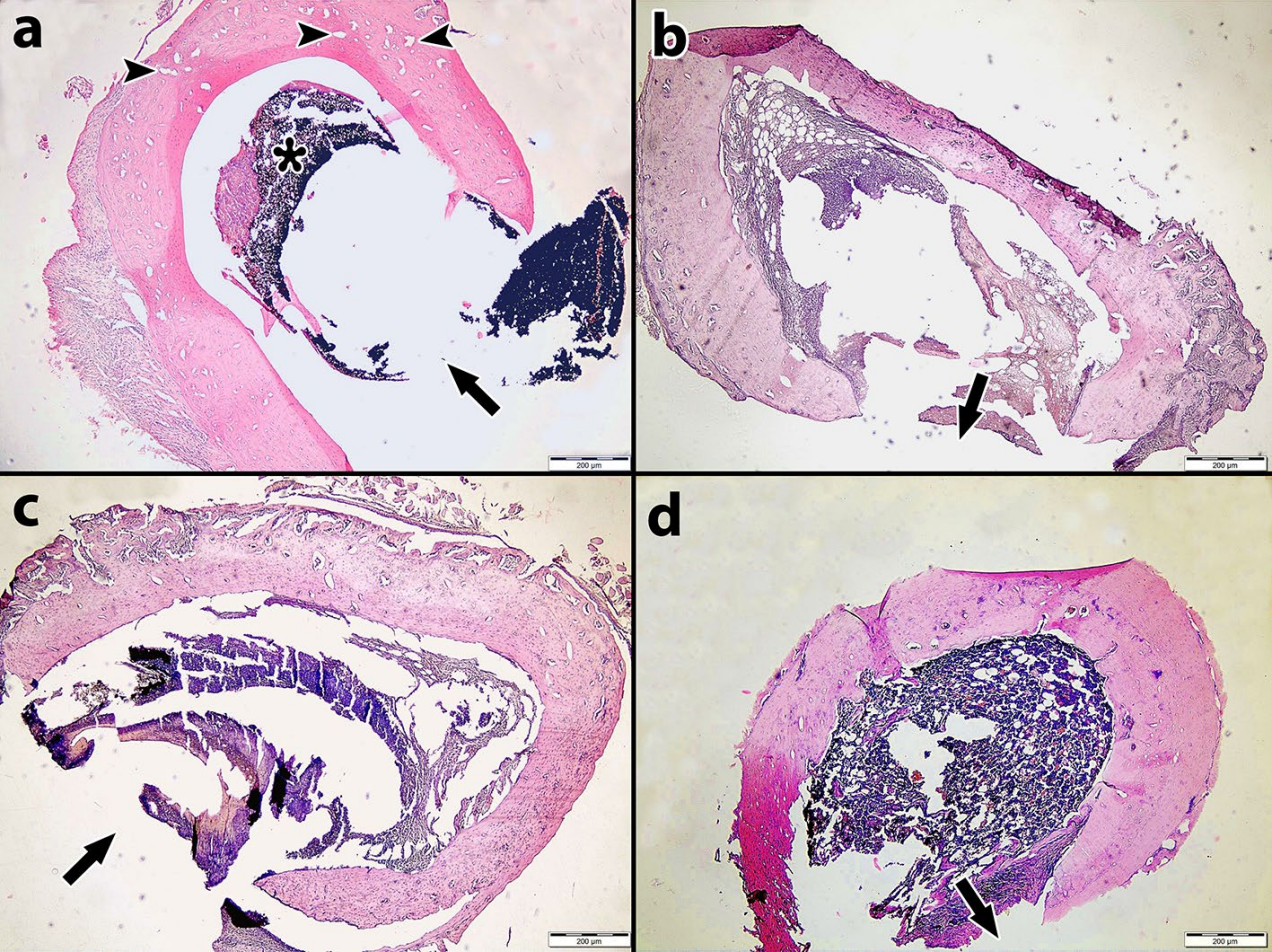
Histological sections of rat femur bone stained with Hematoxylin–Eosin on the 14th day. (**a**) nMTA group; There is a decrease in filling material. Enlargements were detected in the Haversian canals. There is granulation in the bone medulla. No shrinkage was observed in the defect area. (**b**) ERRM group; No filling material was observed, fibrous tissue formation and shrinkage were detected in the defect area. (**c**) ER, no filling material was observed, fibrous tissue was detected in the bone medulla. There is no shrinkage in the defect area. (**d**) Control group; There is dense fibrous tissue increase in the bone medulla. (Magnification bar = 200 µm, H&E 400 ×). Arrow: Defect area, Asterisk: Root end-filling material. (n = 7).

**Figure 5:**
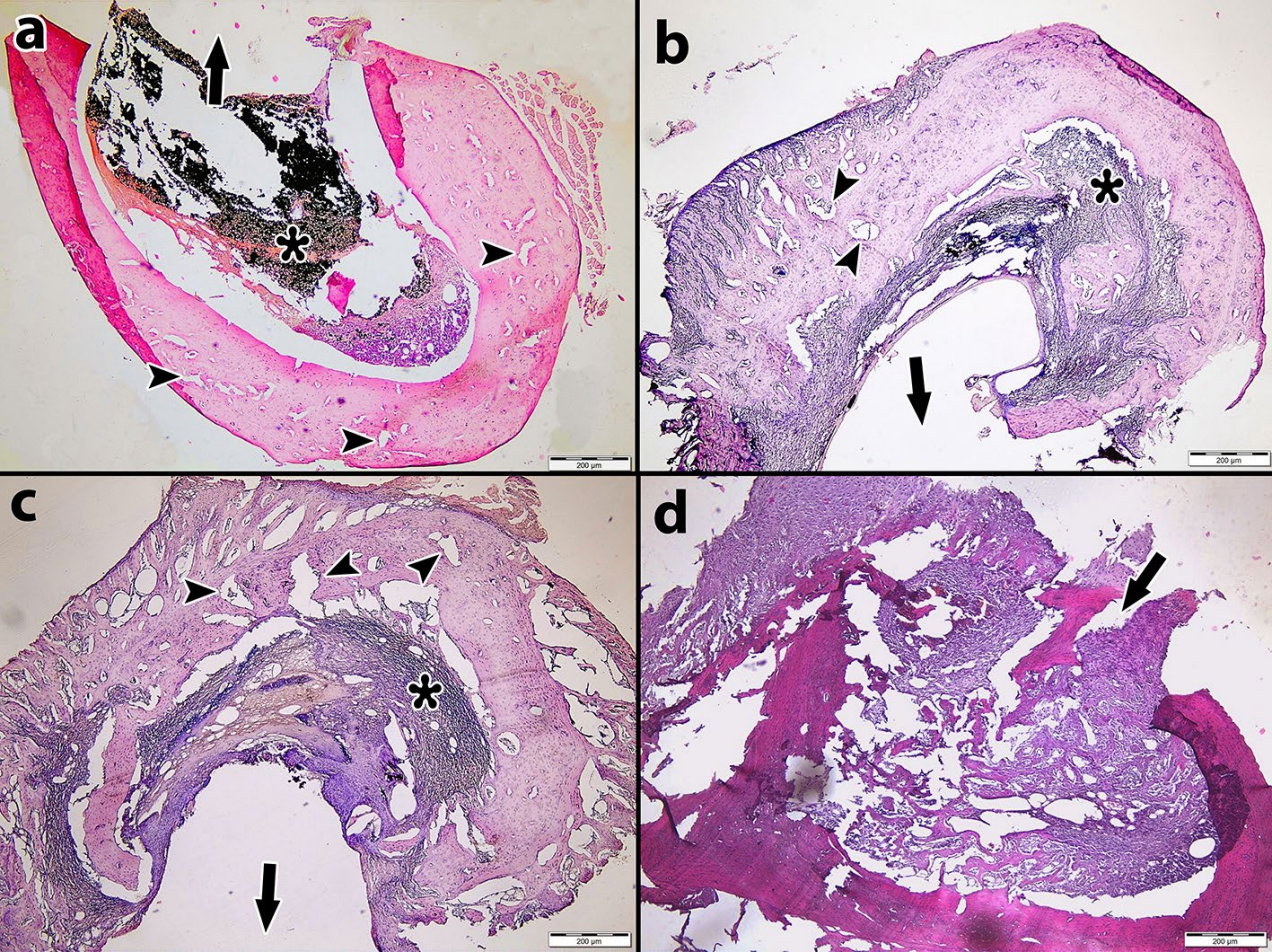
Histological sections of rat femur bone stained with Hematoxylin–Eosin on the 28th day. (**a**) nMTA group; Extensive dilation was detected in the Haversian canals. Filling material available. There is no closure in the defect area. (**b**) ERRM group; There is enlargement of the Haversian canals. Increased fibrous tissue formation was detected in the bone medulla. There is shrinkage in the defect area. (**c**) ER, no filling material was observed, there are intense dilations in the haversian canals. There is increased fibrous tissue in the bone medulla. (**d**) Control group; There are intense deteriorations in the compact structure of the bone with increasing time. (Magnification bar = 200 µm, H&E 400 ×). Arrow: Defect area, Asterisk: Root end-filling material. (n = 7).

The results of the bone healing scores are presented in Table 2, and the distribution of the bone healing scores by the materials and days used is presented in Table 3. Figure 2 illustrates the comparison of the bone healing levels of the materials on different days. Photomicrographs of H&E-stained rat thigh bones are shown in Figs. 3, 4, and 5.

**Table 2:** Bone healing scores of groups on different days.

Day	Material	Min–Max	Avr ± SD	Median	p
**7**	nMTA	3–4	^b^3.5 ± 0.5	4	0.000*
	ERRM	2–3	^a^2.6 ± 0.5	3	
	ER	3–3	^a^3.0 ± 0.0	3	
	Control	3–4	^b^3.5 ± 0.5	3	
**14**	nMTA	3–4	^b^3.3 ± 0.5	3	0.000*
	ERRM	2–3	^a^2.5 ± 0.5	3	
	ER	2–3	^a^2.7 ± 0.4	3	
	Control	2–3	^a^2.5 ± 0.5	3	
**28**	nMTA	2–3	^c^2.7 ± 0.5	3	0.000*
	ERRM	2–3	^ab^2.3 ± 0.4	2	
	ER	1–3	^a^2.1 ± 0.3	2	
	Control	2–3	^bc^2.5 ± 0.5	3	

Kruskal‒Wallis test; post hoc Dunn’s test *p < 0.05.

Different letters in the columns show the difference between materials.

**Table 3:** Percentages of bone healing scores by materials and days.

	BHS	nMTA	ERRM	ER	Control	Total
7th day	1	0 (0.0%)	0 (0.0%)	0 (0.0%)	0 (0.0%)	0 (0.0%)
	2	0 (0.0%)	13 (37.1%)	0 (0.0%)	0 (0.0%)	13 (9.3%)
	3	16 (45.7%)	22 (62.9%)	35 (100%)	19 (54.3%)	92 (65.7%)
	4	19 (54.3%)	0 (0.0%)	0 (0.0%)	16 (45.7%)	35 (25%)
	5	0 (0.0%)	0 (0.0%)	0 (0.0%)	0 (0.0%)	0 (0.0%)
14th day	1	0 (0.0%)	0 (0.0%)	0 (0.0%)	0 (0.0%)	0 (0.0%)
	2	0 (0.0%)	17 (48.6%)	9 (25.7%)	17 (48.6%)	43 (30.7%)
	3	25 (71.4%)	18 (51.4%)	26 (74.3%)	18 (51.4%)	87 (62.1%)
	4	10 (28.6%)	0 (0.0%)	0 (0.0%)	0 (0.0%)	10 (7.1%)
	5	0 (0.0%)	0 (0.0%)	0 (0.0%)	0 (0.0%)	0 (0.0%)
28th day	1	1 (2.9%)	0 (0.0%)	0 (0.0%)	0 (0.0%)	1 (0.7%)
	2	11 (31.4%)	26 (74.3%)	31 (88.6%)	16 (45.7%)	84 (60%)
	3	24 (68.6%)	9 (25.7%)	3 (8.6.0%)	19 (54.3%)	55 (39.3%)
	4	0 (0.0%)	0 (0.0%)	0 (0.0%)	0 (0.0%)	0 (0.0%)
	5	0 (0.0%)	0 (0.0%)	0 (0.0%)	0 (0.0%)	0 (0.0%)

BHS: Bone Healing Score.

### Histopathological results on the 7th day

The results indicated that in 54.3% of the defects treated with nMTA, immature bone formation and new cartilage formation were observed on histopathological examination. Furthermore, new cartilage formation was observed in 62.9% of the defects treated with ERRM, in all defects treated with the ER material, and in 54.3% of the control group, although new bone formation was not observed. Statistical analysis revealed that the bone healing levels of the ERRM and ER groups were significantly lower than those of the nMTA and control groups (p < 0.05). No statistically significant differences were found among the other groups on day 7 (p > 0.05).

### Histopathological results on the 14th day

New cartilage formation in the defects was observed in 71.4%, 51.4%, 74.3%, and 51.4% of the nMTA, ERRM, ER, and control groups, respectively. Statistically significant differences were observed between the nMTA group and the ERRM, ER, and control groups, with the bone healing level of the nMTA group being notably higher (p < 0.05). No statistically significant differences were observed among the other groups on day 14 (p > 0.05).

### Histopathological results on the 28th day

The results of this study indicated that new cartilage formation occurred in 68.6% of the defects in which nMTA material was used and in 54.3% of the control group. However, new bone formation was not observed. Furthermore, inflammation and/or fibrosis were predominant in 74.3% of defects treated with ERRM and 88.6% of defects treated with ER, respectively. The bone healing level in the ERRM group was significantly lower than that in the nMTA group (p < 0.05). Similarly, the bone healing level of the ER group was considerably lower than that of the nMTA and control groups (p < 0.05). No statistically significant differences were observed among the other groups (p > 0.05). Over time, the compact bone organization in all groups deteriorated, with enlargement and irregularities in the Haversian canals observed most on day 28. Granulated tissue formation was observed in the bone medulla on day 28 in the ERRM and ER groups. No necrosis or intense inflammation was detected in the defect areas of any group at any time point (Figs. 3, 4, 5).

## Discussion

Root-end filling materials, commonly used in pediatric dentistry, are in constant contact with the hard and soft tissues in the periapical region. It is essential that these materials, used as apical barriers in single-session apexification therapy in immature permanent teeth, have a high potential to induce hard-tissue formation. To evaluate this potential, toxicity tests, animal studies consisting of connective tissue or intraosseous implants, sensitization, oral mucosa tests, and many methods conducted on humans or animals according to clinical use have been employed^21–23^.

Numerous clinical studies addressing apical plugs are available in the existing literature^24–26^. Given the limited availability of in vitro studies pertaining to ER, one of the dental materials under scrutiny in this investigation, an animal model was employed as an intermediary step prior to embarking on clinical trials^15,16^. One methodology employed to simulate clinical scenarios associated with apical plugs entails the application of such plugs to the periapical regions of animal teeth, as exemplified in histological studies conducted, for instance, in dogs^27,28^. The reason for choosing rats in this study was the low cost, time efficiency, low risk of postoperative infection, homogeneity of the genetic background, and ease of comparing tissue responses in the same animal^29^. Furthermore, the direct placement into surgically created defects within the bone has emerged as a methodology employed to assess the osteoinductive potential of dental materials, a practice aligned with studies employing analogous methodologies^19,30,31^. Our study was conceptualized with a framework centered on femur defects, aligning with similar investigations conducted on the tibia, calvaria, and condylar bones^19,30,31^. We used male rats because they have a genetic structure similar to that of humans, are low in cost, are in the lowest category of readily available animals and are unlikely to affect tissue reactions due to variations in hormone cycles. In the current study, examinations were performed on rat tissues on the 7th, 14th, and 28th days, which allowed results to be obtained in a shorter time compared with studies conducted in rabbits and pigs^18^.

MTA, accepted as the gold standard as a root-end filling material with its anti-inflammatory and osteogenic potential, as well as many favorable properties, has been evaluated in many studies^32–34^. nMTA, which contains tricalcium silicate (alite), dicalcium silicate (belite), calcium sulfate (as anhydrite), and a low amount of calcite that facilitates the formation of calcium hydroxide, has taken its place among the new products as an advanced version of MTA^35^. Although less is known about the biological properties of nMTA, which has a similar composition, studies have shown that it possesses alkaline phosphatase activity, which is essential for high mineral deposition^36^. nMTA was the only group in which no inflammation or fibrosis was observed in any sample during the entire experiment and 100% new cartilage formation occurred. In the defect areas where nMTA was placed, immature bone formation was observed at a rate of 54.3% on the 7th day and 28.6% on the 14th day. The remarkable results of our research on nMTA corroborate those of many studies that have highlighted the superior properties of the material^9,37,38^.

Another calcium silicate-based material known as ERMM, which is available in an injectable form, has been developed to address the drawbacks associated with MTA. It possesses physical properties similar to those of MTA and has been shown to possess additional advantages in terms of usage^39^. Moreover, various studies have been conducted to compare ERMM with MTA in terms of their efficacy for in vitro sealing and regenerative potential in both animal periradicular tissue and human cells^12,40,41^. The results of the current study are in line with those of many other studies demonstrating that ERRM, which does not display a critical cytotoxic profile, has a cytokine-inducing ability similar to that of MTA. The dissimilarity in tissue responses to nMTA can be attributed to the differences in composition. In our study, a predominance of inflammation or fibrosis was observed, at a rate of 37.1% on the 7th day, 48.6% on the 14th day, and 74.3% on the 28th day. Increases in the thickness of fibrous capsules can be attributed to fibrosis, which can be considered a part of the inflammatory process. In another study comparing the subcutaneous implant application of ERRM and MTA with connective tissue reactions, the fact that ERRM caused significantly more tissue irritation and thickening of the fibrous capsule than the control group is similar to the results of our study^42^. ERRM was also found to be significantly more irritating than MTA and the control at weeks one and three but showed higher biocompatibility after six weeks of use. Therefore, it can be concluded that increasing the evaluation time interval may lead to different results.

In our study, it was observed that nMTA, which was observed to have better efficacy in terms of new bone formation in the first week than other materials, showed statistical similarity with the control group formed by the defect areas in which no material was placed, and other material groups remained below this level. In this respect, it is similar to another study that concluded that both ERRM and MTA had a detrimental effect on the tissue when implanted in rat subcutaneous tissue after 7 and 30 days^17^. However, the findings of the study showed that the fibrous capsule thickness in MTA increased significantly compared to that in the ERRM groups, and these necrotic areas were observed more frequently, in contrast to our study. This difference may be due to the differences in the application technique and texture studied, and longer observations will better reveal the real behavior of the materials. Additionally, the use of different updated forms of MTA in different studies is expected to change the results obtained.

To the best of our knowledge, no in vivo or clinical studies have been conducted on ER. However, only two in vitro studies have shown that ER has an effect similar to that of tricalcium phosphate-containing materials in terms of cytotoxicity^15,16^. Our study is the first in vivo study to compare ER with other root-end filling materials. The ER is composed of calcium phosphate, hydroxyapatite, and distilled water^14^. It has been hypothesized that nMTA and ERRM materials may form a hydroxyapatite-like layer when exposed to physiological tissue fluids, whereas ER may exhibit enhanced osteoinductive potential owing to the presence of hydroxyapatite within its structure. This study demonstrated that the expected level of osteoinductivity could not be achieved. The ER values were below those of the control group, particularly in the first week, and there was no statistically significant difference compared to the other groups. Although the difference was not statistically significant, there was no sign of improvement in the defect area in one sample on the 28th day. One of the limitations was the absence of improvement and the occurrence of necrosis in the same scoring category.

As expected, in the control group, the inflammation and fibrosis in the defect area increased over time. However, even if there was no significant difference, especially in the first week, the higher recovery scores compared to the other groups may be attributed to the high turnover rate in animals^29^. One of the limitations of this study was that it did not distinguish between fibrosis and inflammation in the scoring system.

As bone formation was induced in the nMTA group, albeit immature, the null hypothesis that the tested materials could not repair the bone when applied to the cavities created in the femoral rat models was rejected. The second null hypothesis was rejected because there was a difference between the materials in terms of their osteoinductive properties. The small workspace required for extra precision during surgical procedures and the differences between potential reactions in the type of animal tissue used to mimic the hard and soft tissues of the human periodontium are limitations of this study. However, the larger sample size in a more extended observation that goes beyond the reported setting of the test materials and that one of the experimental groups had not been previously studied in animals may have contributed to better research coverage.

This study has certain limitations. Firstly, the limited number of rats in each group is attributed to ethical and financial constraints. Furthermore, the utilization of rats as experimental subjects was prompted by the challenging ethical regulations in our country, which impose restrictions on the use of alternative animals for research purposes. Thirdly, the outcomes of this study are specifically applicable to the rat femur bone defect model, and extrapolating these findings to humans requires caution. Consequently, we leveraged defects created in the femur bones, mimicking the apical regions of teeth, within the selected animal group to address these constraints. Additional experimental and clinical studies may be deemed necessary for a thorough evaluation of the bone healing potential associated with the experimental materials. While the results imply potential benefits in bone healing processes with the use of these root-end filling materials, it is crucial to acknowledge that in vivo evidence for the ER material has not been scrutinized in human clinical trials. Regrettably, positive outcomes observed in animal models do not consistently translate into clinical efficacy in humans. Nevertheless, animal models function as valuable tools in the exploration and quantification of treatment effects, aiding in the comprehension and prediction of responses in humans. Data derived from animal studies play a pivotal role in shaping and guiding the design of potential future human studies.

## Conclusion

Within the limitations of this study, the results of this study showed that nMTA has better osteoinductive properties than other materials. It has been revealed that ERRM and ER materials developed as alternatives to MTA can also be used as root end-filling materials; however, more in vitro and ex vivo studies are needed to evaluate tissue and cellular responses.

## Data Availability

The data that support the findings of this study are available from the corresponding author upon reasonable request.

## References

[1] Duggal M, Tong HJ, Al-Ansary M, Twati W, Day PF, Nazzal H (2017). Interventions for the endodontic management of non-vital traumatised immature permanent anterior teeth in children and adolescents: A systematic review of the evidence and guidelines of the European Academy of Paediatric Dentistry. Eur. Arch. Paediatr. Dent..

[2] Gunes B, Aydinbelge HA (2012). Mineral trioxide aggregate apical plug method for the treatment of nonvital immature permanent maxillary incisors: Three case reports. J. Conserv. Dent..

[3] Solanki NP, Venkappa KK, Shah NC (2018). Biocompatibility and sealing ability of mineral trioxide aggregate and biodentine as root-end filling material: A systematic review. J. Conserv. Dent..

[4] Komabayashi T, Spangberg LSW (2008). Comparative analysis of the particle size and shape of commercially available mineral trioxide aggregates and Portland cement: A study with a flow particle image analyzer. J. Endodont..

[5] Shahi S, Rahimi S, Lotfi M, Yavari H, Gaderian A (2006). A comparative study of the biocompatibility of three root-end filling materials in rat connective tissue. J. Endod..

[6] Parirokh M, Torabinejad M (2010). Mineral trioxide aggregate: a comprehensive literature review–Part III: Clinical applications, drawbacks, and mechanism of action. J Endod..

[7] Camilleri J (2015). Staining potential of neo MTA plus, MTA plus, and biodentine used for pulpotomy procedures. J. Endod..

[8] Srinivasan V, Waterhouse P, Whitworth J (2009). Mineral trioxide aggregate in paediatric dentistry. Int. J. Paediatr. Dent..

[9] Abboud KM, Abu-Seida AM, Hassanien EE, Tawfik HM (2021). Biocompatibility of NeoMTA Plus(R) versus MTA angelus as delayed furcation perforation repair materials in a dog model. BMC Oral Health..

[10] Sogukpinar A, Arikan V (2020). Comparative evaluation of four endodontic biomaterials and calcium hydroxide regarding their effect on fracture resistance of simulated immature teeth. Eur. J. Paediatr. Dent..

[11] Shokouhinejad N, Nekoofar MH, Razmi H, Sajadi S, Davies TE, Saghiri MA (2012). Bioactivity of EndoSequence root repair material and bioaggregate. Int. Endod. J..

[12] Damas BA, Wheater MA, Bringas JS, Hoen MM (2011). Cytotoxicity comparison of mineral trioxide aggregates and EndoSequence bioceramic root repair materials. J. Endod..

[13] Lovato KF, Sedgley CM (2011). Antibacterial activity of endosequence root repair material and proroot MTA against clinical isolates of Enterococcus faecalis. J. Endod..

[14] EN_ Hoffmanns- Catalog- 01. pdf. In.; 2016. https://hoffmann-dental.com/wp-content/uploads/2017/02/EN_Hoffmanns-Catalog-01-2016.pdf

[15] Aksu S, Gürbüz T (2020). Evaluation of total oxidant and antioxidant status of various pulp capping materials on human dental pulp stem cells. Selcuk Dent. J..

[16] Delikan E, Aksu S (2020). Comparison of the sealing ability of apical plug materials in simulated open apices: An in vitro study. J. Oral. Res. Rev..

[17] Khalil WA, Abunasef SK (2015). Can mineral trioxide aggregate and nanoparticulate EndoSequence root repair material produce injurious effects to rat subcutaneous tissues?. J. Endod..

[18] Scelza MZ, Campos CA, Scelza P, Adeodato CS, Barbosa IB, de Noronha F (2016). Evaluation of inflammatory response to endodontic sealers in a bone defect animal model. J. Contemp. Dent. Pract..

[19] Akhavan A, Parashos P, Razavi SM, Davoudi A, Shadmehr E (2017). Hard tissue reaction to mineral trioxide aggregate and experimental root-end filling material in guinea pig mandibles. J. Dent. Sci..

[20] Cintra LT, de Moraes IG, Estrada BP, Gomes-Filho JE, Bramante CM, Garcia RB (2006). Evaluation of the tissue response to MTA and MBPC: Microscopic analysis of implants in alveolar bone of rats. J. Endod..

[21] Bodrumlu E (2008). Biocompatibility of retrograde root filling materials: A review. Aust Endod. J.

[22] Santos JM, Coelho CM, Sequeira DB, Marques JA, Pereira JF, Sousa V (2021). Subcutaneous implantation assessment of new calcium-silicate based sealer for warm obturation. Biomedicines..

[23] Simsek N, Alan H, Ahmetoglu F, Taslidere E, Bulut ET, Keles A (2015). Assessment of the biocompatibility of mineral trioxide aggregate, bioaggregate, and biodentine in the subcutaneous tissue of rats. Niger J. Clin. Pract..

[24] Burns LE, Gencerliler N, Terlizzi K, Solis-Roman C, Sigurdsson A, Gold HT (2023). Apexification outcomes in the United States: A retrospective cohort study. J. Endod..

[25] Ree MH, Schwartz RS (2017). Long-term success of nonvital, immature permanent incisors treated with a mineral trioxide aggregate plug and adhesive restorations: A case series from a private endodontic practice. J Endod..

[26] Santos JM, Diogo P, Dias S, Marques JA, Palma PJ, Ramos JC (2022). Long-term outcome of nonvital immature permanent teeth treated with apexification and corono-radicular adhesive restoration: A case series. J Endod..

[27] Palma PJ, Ramos JC, Martins JB, Diogenes A, Figueiredo MH, Ferreira P (2017). Histologic evaluation of regenerative endodontic procedures with the use of chitosan scaffolds in immature dog teeth with apical periodontitis. J. Endod..

[28] Stambolsky C, Rodriguez-Benitez S, Gutierrez-Perez JL, Torres-Lagares D, Martin-Gonzalez J, Segura-Egea JJ (2016). Histologic characterization of regenerated tissues after pulp revascularization of immature dog teeth with apical periodontitis using tri-antibiotic paste and platelet-rich plasma. Arch. Oral Biol..

[29] Ozbas H, Yaltirik M, Bilgic B, Issever H (2003). Reactions of connective tissue to compomers, composite and amalgam root-end filling materials. Int Endod J..

[30] Gandolfi MG, Iezzi G, Piattelli A, Prati C, Scarano A (2017). Osteoinductive potential andp bone-bonding ability of ProRoot MTA, MTA Plus and Biodentine in rabbit intramedullary model: Microchemical characterization and histological analysis. Dent Mater..

[31] Xu S, Lin K, Wang Z, Chang J, Wang L, Lu J (2008). Reconstruction of calvarial defect of rabbits using porous calcium silicate bioactive ceramics. Biomaterials.

[32] Liu M, He L, Wang H, Su W, Li H (2021). Comparison of in vitro biocompatibility and antibacterial activity of two calcium silicate-based materials. J. Mater. Sci. Mater. Med..

[33] Rodrigues EM, Viola KS, Maldonado LG, Rossa Junior C, Guerreiro-Tanomaru JM, Tanomaru FM (2022). Cytotoxicity and bioactive potential of new root repair materials for use with BMP-2 transfected human osteoblast cells. Braz Oral. Res..

[34] Rodriguez-Lozano FJ, Lozano A, Lopez-Garcia S, Garcia-Bernal D, Sanz JL, Guerrero-Girones J (2022). Biomineralization potential and biological properties of a new tantalum oxide (Ta(2)O(5))-containing calcium silicate cement. Clin. Oral Investig..

[35] Hoshino RA, Delfino MM, da Silva GF, Guerreiro-Tanomaru JM, Tanomaru-Filho M, Sasso-Cerri E (2021). Biocompatibility and bioactive potential of the NeoMTA Plus endodontic bioceramic-based sealer. Restor. Dent. Endod..

[36] Tanomaru-Filho M, Andrade AS, Rodrigues EM, Viola KS, Faria G, Camilleri J (2017). Biocompatibility and mineralized nodule formation of Neo MTA Plus and an experimental tricalcium silicate cement containing tantalum oxide. Int. Endod. J..

[37] Sismanoglu S, Ercal P (2023). Effects of calcium silicate-based cements on odonto/osteogenic differentiation potential in mesenchymal stem cells. Aust. Endod. J..

[38] Walsh RM, Woodmansey KF, He J, Svoboda KK, Primus CM, Opperman LA (2018). Histology of NeoMTA plus and Quick-Set2 in contact with pulp and periradicular tissues in a canine model. J Endod..

[39] Walsh RM, Woodmansey KF, Glickman GN, He J (2014). Evaluation of compressive strength of hydraulic silicate-based root-end filling materials. J. Endod..

[40] Ciasca M, Aminoshariae A, Jin G, Montagnese T, Mickel A (2012). A comparison of the cytotoxicity and proinflammatory cytokine production of EndoSequence root repair material and ProRoot mineral trioxide aggregate in human osteoblast cell culture using reverse-transcriptase polymerase chain reaction. J. Endod..

[41] Rifaey HS, Villa M, Zhu Q, Wang YH, Safavi K, Chen IP (2016). Comparison of the osteogenic potential of mineral trioxide aggregate and endosequence root repair material in a 3-dimensional culture system. J. Endod..

[42] Taha NA, Safadi RA, Alwedaie MS (2016). Biocompatibility evaluation of EndoSequence root repair paste in the connective tissue of rats. J Endod..

